# Strength of evidence for paternal influence on offspring epigenome in observational human studies: a systematic review and risk-of-bias appraisal for non-randomized exposures

**DOI:** 10.1080/15592294.2025.2594322

**Published:** 2025-12-10

**Authors:** Ka Kei Sum, Mark A. Burton, Cristina Garcia-Maurino Alcazar, Ai Ling Teh, Keith M. Godfrey, Jonathan Yinhao Huang

**Affiliations:** a Institute for Human Development and Potential, Agency for Science, Technology and Research, Singapore, Singapore; b Population Health Sciences, Bristol Medical School, University of Bristol, Bristol, UK; c Human Development and Health Academic Unit, Faculty of Medicine, University of Southampton, Southampton, UK; d Institute for Global Health, University College London, London, UK; e Bioinformatics Institute, Agency for Science, Technology and Research, Singapore, Singapore; f MRC Lifecourse Epidemiology Centre, University of Southampton, Southampton, UK; g NIHR Southampton Biomedical Research Centre, University Hospital Southampton NHS Foundation Trust and University of Southampton, Southampton, UK; h Department of Public Health Sciences, Thompson School of Social Work & Public Health, University of Hawaiʻi at Mānoa, Honolulu, Hawaiʻi, USA

**Keywords:** Epigenetics, paternal effects, causal inference, molecular epidemiology

## Abstract

Paternal influence on the offspring epigenome is difficult to interpret in observational human studies due to heterogeneous designs, causing varying susceptibility to bias and non-causal explanations. We conducted a systematic review (CRD42022302695) of paternal exposures before or during pregnancy in relation to the offspring epigenome, focusing on characteristics affecting causal interpretation and reproducibility. We searched three electronic databases for human studies published between 2003 and 2023. Eligible studies assessed paternal factors before or during pregnancy as exposures, and epigenetic mechanisms as outcomes. Risk of bias (RoB) was evaluated using ROBINS-E. The most frequently studied paternal factors were BMI/obesity (7), age (7), smoking (5), and socioeconomic status (SES) (4). All 28 studies assessed DNA methylation; two additionally explored miRNA expression. Most studies were rated ‘high’ or ‘very high’ RoB, primarily due to unclear exposure measurement and confounding. Findings showed limited overlap in CpG sites and genomic regions across studies. However, exposures that were stable (*e.g*. SES) or had clearly defined timing produced more consistent results. Notably, studies with clearly defined timing of paternal smoking suggested preconception exposure may influence offspring epigenetic pathways related to innate immunity but not pregnancy exposure. In contrast, paternal factors with poorly defined experimental analogues, such as BMI, provided inconsistent results. Aligning study design more closely with clinical trials or animal models, by clearly defining populations and exposures, may result in more reliable and replicable findings. Frameworks like ‘target trial emulation,’ offer a promising approach to improve reproducibility and interpretability of future research on paternal effects on offspring epigenome.

## Introduction

Assumptions regarding the causal primacy of maternal effects on child health have shaped the landscape of Developmental Origins of Health and Disease (DOHaD) research since the hypothesis was championed more than 30 years ago by Barker and Osmond [1]. While it is known that both parents contribute equally to a child’s genome, paternal environmental influences on child health, specifically surrounding the peri-conceptional period, remain largely understudied compared with maternal influences. Although studies of fathers during pregnancy and childbirth started as early as the 1960 s by social scientists, they were mostly descriptive in nature. Some of the work involved understanding the emotions of expectant fathers [2] and the impact of their absence or presence during childbirth [3]. Only in recent years has evidence from animal experimental studies started to provide insights into the potential mechanisms underlying paternally transmitted effects [4,5].

**Figure 1.**
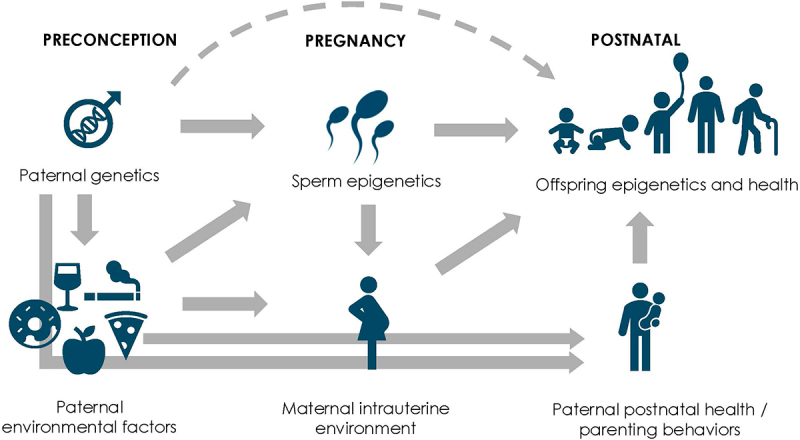
Diagram demonstrating potential mechanisms involved in the intergenerational transmission of paternal factors on offspring health.

Systematic reviews in 2018 [6] and 2020 [7] identified some evidence of associations between paternal factors around conception (i.e., age, body mass index (BMI), birth weight, smoking, etc.) and a range of adverse psychiatric disorders and physical health outcomes in the offspring, including cardiovascular and metabolic health outcomes. Various mechanisms have been proposed to explain the effect of paternal exposure on offspring health (Figure 1). These include direct genetic transmission of alleles or epigenetic markers such as DNA methylation, non-coding RNAs (ncRNAs), and histone modifications via sperm factors or seminal plasma [8], and mediation by epigenetic processes, defined as ‘those that stably affect gene expression through mechanisms not involving the primary nucleotide sequence’ [9]. This is supported by experimental studies that have linked various epigenetic manipulations or changes to paternal environmental perturbations, including dietary interventions [10,11], psychological stress [12], and endocrine disruptors [13]. However, questions remain as to whether changes in sperm epigenetics translate into epigenetic changes in the offspring and thus affect offspring health outcomes. Other indirect mechanisms involve moderation or mediation by maternal responses, where fathers influence maternal health or health behaviors during pregnancy [14–17], altering the offspring’s epigenome and health outcomes.

Owing to the multiplicity of potential pathways as to how fathers may influence offspring health, good study and analytic designs are especially crucial in disentangling causal from non-causal associations in observational studies. Current evidence surrounding the association between paternal factors and the offspring epigenome in observational human studies has been inconsistent, including results from targeted analysis of imprinted regions and Epigenome-wide Association Studies (EWAS). Such studies often fail to consider elements that consist of a good study design (*i.e*., confounding, measurement error, etc.) and incorporate relevant data and analytic strategies [18,19]. While analytical and statistical strategies used to process epigenetic biomarker data, such as techniques used for normalization, cell-type correction, and multiple testing, may affect heterogeneity in findings, studies [20,21] have previously shown that heterogeneity attributed to analytical and statistical strategies is minimal, and appropriate parameters used to evaluate study design matter more substantively in producing consistent and interpretable results. As epigenetic outcomes are known to be malleable to perturbations at specific sensitive windows of exposure, specificity in exposure definition, such as timing, is also instrumental in uncovering potential underlying mechanisms that may be involved in the observed associations and biases. For example, we may not replicate epigenetic signals observed in studies that measured paternal smoking exposure postnatally compared to those measured prenatally, because the underlying mechanisms are essentially distinct at different times. In other instances, we may observe replication in both timings simply due to the association between postnatal and prenatal exposure, instead of true persistent effects. To better understand how paternal factors may affect offspring epigenome and thus offspring health, we aimed to assess the overall strength of evidence in the current literature associating various peri-conceptional paternal factors with epigenetic changes measured within the offspring at all ages by focusing on important study design parameters using a tool developed under the target trial framework, Risk Of Bias In Non-randomized Studies of Exposures (ROBINS-E) [22]. The target trial framework [23] is a method developed to formalize the specification of the effect of exposure on outcome and to estimate such effects without bias by carefully considering the elements that make a successful trial, such as a well-defined study population (start point and length of follow-up), exposure (timing, dose, and duration of exposure), and outcome measurement. We suggest that the use of such a risk-of-bias evaluation tool would help us identify the sources of major biases in the current literature that contribute to the lack of interpretability and replicability of epigenetic findings and hopefully provide guidance for future research in the field. We discuss their correspondence to the existing body of animal experimental findings, which are less susceptible to the biases of conventionally analyzed human studies, and how together these designs can inform future research directions.

## Methods

This systematic review was conducted and reported according to the Preferred Reporting Items for Systematic Reviews and Meta-analyses (PRISMA) guidelines (S1 Appendix). The protocol for our systematic review was registered in the International Prospective Register of Ongoing Systematic Reviews (PROSPERO; CRD42022302695; S2 Appendix).

### Literature searches and databases

We searched three electronic databases (Embase, Medline, and Cochrane) for relevant articles published in the English language between 1 January 2003 and 7 January 2023. We included publications starting in 2003 due to the emergence of next-generation sequencing and microarray-based technologies for analyzing DNA methylation to avoid comparison with earlier studies that lack relevant technologies [24]. Studies that evaluated paternal factors that occurred before and during pregnancy as the exposure of interest and all relevant epigenetic marks/mechanisms (DNA methylation, histone modifications, and ncRNA expression) measured at any time point in the offspring were included as outcomes. For studies in which paternal factors were measured postnatally, we included them only if the authors clearly stated that the measures were retrospective reports of preconception or prenatal exposures, and if the factors were relatively stable over time (*e.g*., education). We excluded studies with postnatal paternal measurements if the exposure window was unclear or clearly postnatal, or the factors were likely to change over time (*e.g*., smoking). Animal studies and studies that did not implement a quantitative approach to summarize the findings (qualitative, narrative reviews, editorials, commentary, and case reports) were excluded.

### Search strategy

A search strategy regarding the selection of MeSH terms and keywords was discussed with an experienced librarian to maximize the sensitivity in the inclusion of all relevant studies. The initial search strategy was piloted to ensure that relevant studies were identified before it was finalized. Keywords used in the searches were based on two broad concepts, ‘paternal exposures’ and ‘epigenetics.’ See S3 Appendix: Table S1-S3 for the full search strategy. The first search was conducted on 18 November 2021. An updated search was conducted on 7 January 2023, using the same search strategy.

### Screening and data extraction

All references identified from the initial database search were managed using Mendeley software. Two reviewers (KKS and MB) independently screened the titles and abstracts using the Rayyan web application [25], followed by full-text screening. Any discrepancies between the two reviewers were discussed to reach consensus on the inclusion of the final articles. The first reviewer (KKS) performed data extraction for all articles using a customized data extraction form that included all the predefined data items published on PROSPERO and compared it with 50% of the information extracted by the second reviewer (MB).

### Strategy for data synthesis

The main results were organized according to paternal exposure type, epigenome outcome (type, tissue, global/targeted/genome-wide epigenetic analysis), and offspring developmental stage. The effect directionality, CpG sites, and annotated genes of epigenetic markers were summarized across all studies. Effect estimates were not combined owing to heterogeneity in setting, exposure, outcome measurement, and risk of bias. We discuss this further below.

### Assessment of methodological quality

Two reviewers (KKS and CGM) independently assessed the quality of the included studies using the Risk Of Bias In Non-randomized Studies- of Exposures (ROBINS-E) tool [22], and discrepancies were discussed and resolved between the two reviewers. Following the guidelines of ROBINS-E, we evaluated the quality of the studies across seven domains: confounding, exposure measurement, selection of participants, post-exposure intervention, missing data, selection of results, and outcome measurement. We used the tool’s signalling questions and embedded automated rule assignments in the Excel template (downloadable from the ROBINS-E website) [26] to determine the risk of bias (RoB) for each domain. Then, we summarized the findings across the seven domains to reach an overall rating (low, some concerns, high, very high) for the risk of bias (RoB) of each study. We also assigned a score to each rating (low = 1, some concerns = 2, high = 3, very high = 4) and summarized the results by means and standard deviations to evaluate the overall performance of each domain.

For certain domains, we developed tailored considerations for assessing RoB specific to our research question. In the confounding domain, we identified ‘common covariates’ that have been shown to influence epigenetic biomarker measurements [27–29], including population structure (genetic ancestry, race, or ethnicity), offspring sex, cell type, technical covariates such as batch and hidden artifacts [30]. Given the challenges in comprehensively defining all relevant covariates for each exposure, we defined a list of exposure-specific covariates that should be minimally included in analyses. These include the corresponding maternal factor, parental SES, and some paternal characteristics that were associated with both the exposure and outcome (S4 Appendix: Excel Table S3). We also reported whether the genetic ancestry, race, or ethnicity of the study sample was specified for each study and whether they were accounted for in the analysis, as population structure may have less impact on epigenetic measurement if the study sample is from a homogenous ancestry (S4 Appendix: Excel Table S2). Following the protocol specified in ROBINS-E [22], studies for whom we could not assess the degree to which they considered confounding were automatically assigned ‘very high’ overall risk and no other domain-specific ratings were given. Studies that did not sufficiently adjust for the list of ‘common covariates’ were assigned a rating of ‘high’ RoB. Finally, studies that sufficiently controlled for common covariates along with some paternal characteristics were assigned a rating of ‘some concerns.’

In the exposure measurement domain, we established three criteria to determine whether an exposure was well-defined, corresponding to the first signalling question: (1) alignment between the measurement timing and relevant exposure window, (2) validity of the measured exposure as a proxy for the ‘true’ underlying exposure, (3) specification of the dose or duration of exposure. A combination of signalling questions – addressing exposure definition, and the presence of differential or non-differential measurement error in the tool – was used to determine the final RoB rating for this domain. A ‘very high’ RoB was assigned to studies that failed to align the timing of measurement with the relevant exposure window, used proxy measures that reflected different mechanisms, did not specify dose or duration, and were likely to introduce differential measurement error. A ‘high’ RoB was assigned when some aspects of the three criteria were met, but the exposure was potentially misclassified, although without introducing differential error. A ‘low’ RoB was assigned was assigned when all three criteria were satisfied and the likelihood of measurement error was low.

Since most epigenetics studies were typically conducted on the sub cohort of a larger cohort based on the availability of DNA methylation data, we also evaluated whether the authors reported the comparison of participant characteristics between the sub cohort and the main cohort for the selection domain. The signalling questions in this domain primarily assessed whether follow-up began at the start of the exposure window of interest, whether participants were included based on characteristics measured after the start of the exposure window, and whether any attempts were made to correct for selection bias. The majority of the studies did not report sufficient information to fully address certain signalling questions, resulting in a RoB rating of ‘some concerns.’ A study with ‘low’ RoB would have follow-up starting at the beginning of the exposure window, a constant effect of exposure over the exposure window, and would not have selection based on participant characteristics measured after the start of the exposure window or would address selection bias using appropriate statistical methods.

Regarding outcome measurement, we considered whether batch effects related to the exposure were assessed. This a pertinent issue in measuring molecular outcomes as batch effects that are highly correlated with the exposure can make it challenging to assess whether observed differences reflect true biological variation or artefacts from batch processing. In such cases, adjusting for batch may inadvertently remove the biological effect of interest. The signalling question also assessed whether outcome assessment could have been influenced by the knowledge of participants exposure history. Since most studies did not report on the association between batch effects and the exposure of interest, and most sample processing was done by technicians blinded to the research questions, most studies were assigned a RoB of ‘some concerns.’

## Results

### Search results

**Figure 2.**
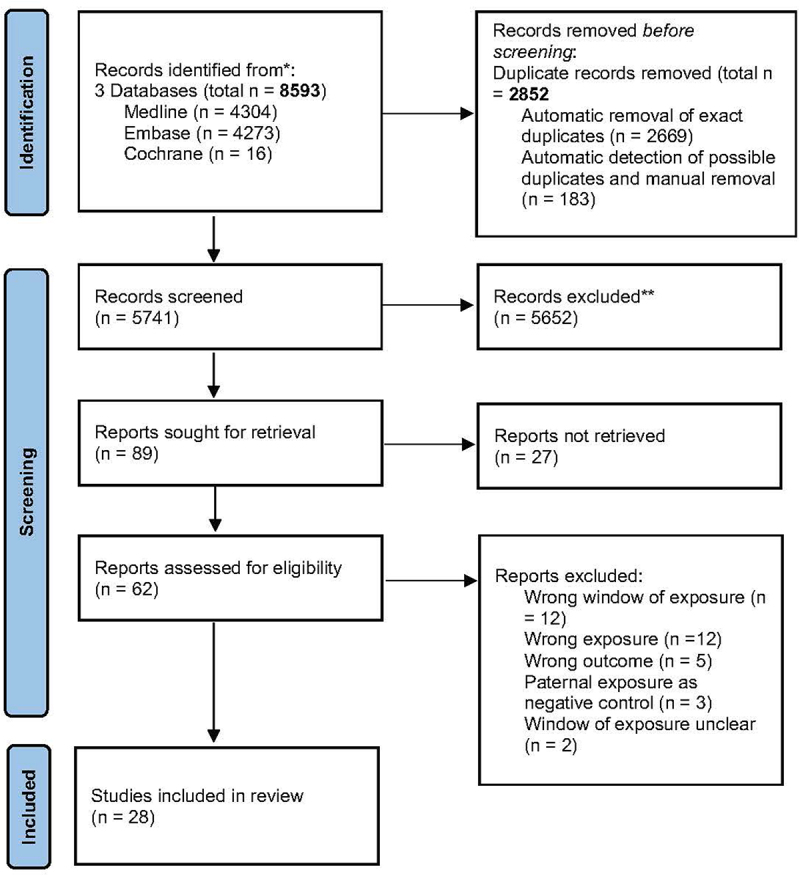
Flow diagram for study selection according to prisma guidelines.

A total of 8593 titles were identified from the three databases combined, with 7932 titles identified from the original search and 661 from the updated search (Figure 2). After removing 2852 articles from duplicates, 5741 abstracts were screened. Eighty-nine articles remained after excluding 5652 articles based on abstract screening, and 62 were successfully retrieved and assessed for eligibility. Of these 62, 34 were excluded after full-text screening, with the main reasons for exclusion being mismatches in the window of exposure of interest (*i.e*., exposure was measured postnatally without clear mention of preconception or pregnancy effects being the main study interest; *N* = 12), paternal exposure not measured (*N* = 12), offspring epigenetic outcome not measured (*N* = 5), paternal exposure as negative control (*N* = 3), and unclear window of exposure (*N* = 2; Figure 2). We excluded studies in which paternal exposure was used exclusively as a negative control, because they were primarily interested in detecting intrauterine effects rather than understanding the contribution of paternal exposure to the offspring epigenome. Further details of the exclusions are included in S4 Appendix: Excel Table S1. Twenty-eight articles were included in the final review.

### Study characteristics

Of the 28 studies included in this review, 24 used data from Europe and America. Twenty-two (78.6%) studies used data from prospective cohorts [31–52], two (7.1%) from retrospective cohorts [53,54], three (10.7%) from cross-sectional studies [55–57], and one (3.6%) from a nested case-control study [58] (Table 1; S4 Appendix: Excel Table S2).

**Table 1.** Characteristics of the 28 studies included.

Study characteristics	Categories	No. of studies (%)^a^	References
Total		28 (100)	
Region	Americas	10 (35.71)	[31,32,35,36,38,40,45,49,53,54]
	Europe	15 (53.57)	[33,34,37,39–41,44,48,50–52,55–58]
	Asia Pacific	3 (10.71)	[42,43,46]
	Middle East/Africa	1 (3.57)	[47]
Type of study	Prospective cohort	22 (78.57)	[31–52]
	Retrospective cohort	2 (7.14)	[53,54]
	Cross-sectional	3 (10.71)	[55–57]
	Nested case-control	1 (3.57)	[58]
Paternal exposure	Age	7 (25.00)	[31–33,53,55–57]
	Ethnicity	1 (3.57)	[45]
	Socioeconomic status (SES)	4 (14.29)	[47–50]
	Atopy history	1 (3.57)	[34]
	Obesity/BMI	7 (25.00)	[35–40,56]
	Smoking	5 (17.86)	[41–44,58]
	Alcohol	1 (3.57)	[46]
	Methyl-group donor intake	1 (3.57)	[51]
	Prenatal attachment and psychopathological symptomatology	1 (3.57)	[52]
	Anthropometrics (weight and height)	1 (3.57)	[39]
	Adverse Child Experiences	1 (3.57)	[54]
Epigenome outcome	Methylation	27 (96.43)	[31–48,50–58]
	Hydroxymethylation	1 (3.57)	[51]
	miRNA	2 (7.14)	[39,57]
	Epigenetic age acceleration	1 (3.57)	[49]
Offspring developmental stage	Delivery	21 (75)	[31,32,34–42,44–46,48–51,55,56,58]
	Early childhood (birth-5 years)	5 (17.86)	[38,40,49,52,54]
	Mid-childhood (6–12 years)	5 (17.86)	[38,40,42,48,49]
	Adolescence (13–18 years)	3 (10.71)	[43,48,49]
	Adulthood ( > 18 years)	5 (17.86)	[33,47,49,53,57]
Tissue type	Cord blood	19 (67.86)	[31,34–38,40–42,44–46,48–51,55,56,58]
	Peripheral blood/whole blood/buffy coat	11 (39.29)	[33,38,40,42,43,47–49,53,54,57]
	Placenta	2 (7.14)	[32,39]
	Buccal swab	2 (7.14)	[49,52]
Analytic approach	Genome-wide	14 (50)	[31–33,38,40,41,43,44,48–50,53,54,57]
	Targeted/candidate gene	15 (53.57)	[34–37,39,42,45–47,49,51,55–58]
	Global	2 (7.14)	[51,52]
Maternal effects considered	Qualitative comparison	13 (46.43)	[31,33–35,37,39,40,47,48,50,52,55,56]
	Adjusted/stratified/restriction	14 (50)	[32,35–38,40,41,44,45,50,53,54,57,58]
	Joint analysis	2 (7.14)	[34,46]
	No	3 (10.71)	[42,43,49]
Functional analyses (omics; GSEA)	Yes	9 (32.14)	[31,33,38–40,42,54,55,57]
	No	17 (60.71)	[32,34–37,41,44–48,50–53,56,58]
	Not applicable^b^	2 (7.14)	[43,49]
Validation	Yes	19 (67.86)	[33–40,42,44,47,49–52,54,55,57,58]
	No	9 (32.14)	[31,32,41,43,45,46,48,53,56]
Replication	Yes	1 (3.57)	[40]
	No	27 (96.43)	[31–39,41–58]

^a^Some variables did not add up to 28 because of non-exclusive categories in some studies. See S4 Appendix: Excel Table S2 for the full details of the characteristics of each study.

^b^Rauschert et al. did not detect any genome-wide significant CpGs; Joyce et al. had epigenetic aging as the outcome.

#### Exposure measurement

The most commonly studied paternal exposures were age [31–33,53,55–57] (*n* = 7) and obesity or body mass index (BMI) [35–40,56] (*n* = 7). This was followed by smoking [41–44,58] (*n* = 5) and socioeconomic status [47–50] (SES; *n* = 4). The remainder of the seven studies focused on exposures such as ethnicity [45] (*n* = 1), atopy history [34] (*n* = 1), alcohol intake [46] (*n* = 1), methyl-group donor intake [51] (*n* = 1), prenatal attachment and psychopathological symptomatology [52] (*n* = 1), anthropometrics [39] (*n* = 1), and adverse childhood experiences [54] (*n* = 1).

#### Outcome measurement

The majority (27/28) of the studies measured DNA methylation as the offspring epigenetic outcome. One study [49] measured epigenetic age acceleration (EAA), and three other studies measuring methylation additionally measured hydroxymethylation [51] and miRNA [39,57]. In terms of the timing of epigenetic outcome measurements, 21 studies were interested in the delivery time frame. Among these 21 studies, five studies [38,40,42,48,49] additionally measured epigenetic outcomes in other developmental stages, including early (0–5 years), mid-childhood (6–12 years), adolescence (13–18 years), and adulthood (18 years and above). Nineteen of the 21 studies measured epigenetic outcomes in cord blood and two [32,39] in the placenta. Peripheral blood, whole blood, and buffy coat (11 studies) were the most common tissues of choice for epigenetic outcome measurement in developmental stages beyond the perinatal period, together with buccal swabs (two studies) [49,52]. Approximately half of the studies focused on targeted or candidate gene approaches, while the other half focused on array-based genome-wide approaches, with only two studies [51,52] assessing global measures. See S4 Appendix: Excel Table S2 for additional study characteristics.

#### Analytic considerations

Half of the studies considered corresponding maternal effects by adjustment, stratification, or restriction. The other half qualitatively compared maternal and paternal estimates by including maternal exposure as an independent variable in a separate model. Four studies [35,37,40,50] performed both adjustment and qualitative comparison of the results. Two studies [34,46] performed joint analysis of both paternal and maternal variables by combining them into a single variable, and one study [34] among the two also performed qualitative comparison of results. Three studies [42,43,49] did not consider maternal effects in their analysis.

To understand the functional and biological mechanisms of epigenetic markers, incorporating other omics technologies (transcriptomics, proteomics, metabolomics), functional annotation, or gene set enrichment analysis (GSEA) into the analysis pipeline is recommended [59,60]. GSEA examines whether a set of genes is enriched in specific biological pathways and their association with the phenotype of interest. Nine studies [31,33,38–40,42,54,55,57] in our review included other forms of omics technologies, functional annotations, or GSEA in their follow-up analyses.

Replication or validation analyses are important for epigenetic studies to confirm the preliminary findings. Replication is defined as ‘the reproducibility of preliminary results in a cohort that is as similar to, but independent of, the preliminary cohort,’ while validation is defined as ‘corroboration of results in a cohort, or using a dataset, that does not originate from the discovery phase of the study’ [60]. Campagna et al. [60] suggested ways to validate their findings when the replication cohort could not be identified. These validation strategies include ‘corroborating preliminary findings in a similar, although not identical cohort, or general population; confirming that preliminary findings are not corroborated in a healthy or natural history cohort or dataset, indicating disease specificity; utilizing EWAS databases to access raw.idat files and/or summary statistics for validation analyses; using the literature to provide biological or pathological support for preliminary results; using animal studies to gain specific mechanistic insight.’ One study [40] performed replication in multiple cohorts within the PACE consortium and meta-analyzed the results. Among the 19 studies that performed some form of validation, one study [49] performed validation in three cohorts, another study [57] used animal experiments to investigate mechanical insights, and the remaining studies mainly compared their results with existing human or animal studies and suggested biological mechanisms based on previous literature in their discussion section.

### Risk of bias assessment

**Figure 3.**
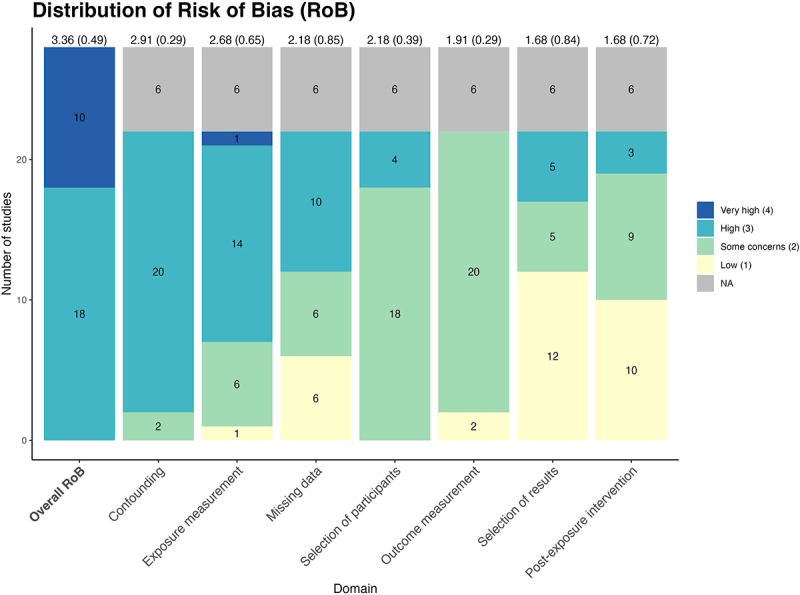
Distribution of risk of bias (RoB) grouped by each domain measured in ROBINS-E. Mean and SD of the RoB quantitative scores were displayed on top of each column. Very high = 4, High = 3, some concerns = 2, and Low = 1.‘NA’ was assigned for the six studies that were not assessed for domain specific RoB because they were automatically assigned to ‘very high’ RoB on the basis that the authors did not consider confounding in their model per protocol set out by ROBINS-E.

All studies included in the review were rated ‘high’ (18 studies) or ‘very high’ (10 studies) for the overall Risk of Bias (RoB; Figure 3). Six studies were automatically assigned ‘very high’ for overall RoB according to the ROBINS-E protocol due to the lack of consideration or exclusion of confounders in these analyses. This resulted in only 22 studies being assessed for domain-specific RoB. After summarizing the overall performance of each domain by taking the mean and standard deviation across studies, most studies had a high risk of bias in the following four domains: confounding (mean = 2.91, SD = 0.29), exposure measurement (mean = 2.68, SD = 0.65), missing data (mean = 2.18, SD = 0.85), and selection of participants (mean = 2.18, SD = 0.39).

According to the ROBINS-E protocol, six studies [31,39,42,45,46,52] that did not consider confounding at all would automatically be assigned a ‘very high’ overall RoB, as previously mentioned. Among the 22 studies that were evaluated for domain-specific RoB, all were rated as ‘high’ RoB except two studies [40,47] were rated ‘some concerns’ for the confounding domain, where both specified the necessary a priori causal pathways regarding potential confounders and mediators. While both studies attempted to control for confounding factors specified in the causal diagrams, certain covariates or paternal characteristics with less substantial impact were not fully accounted for. Thus, they did not qualify for better ratings.

**Figure 4.**
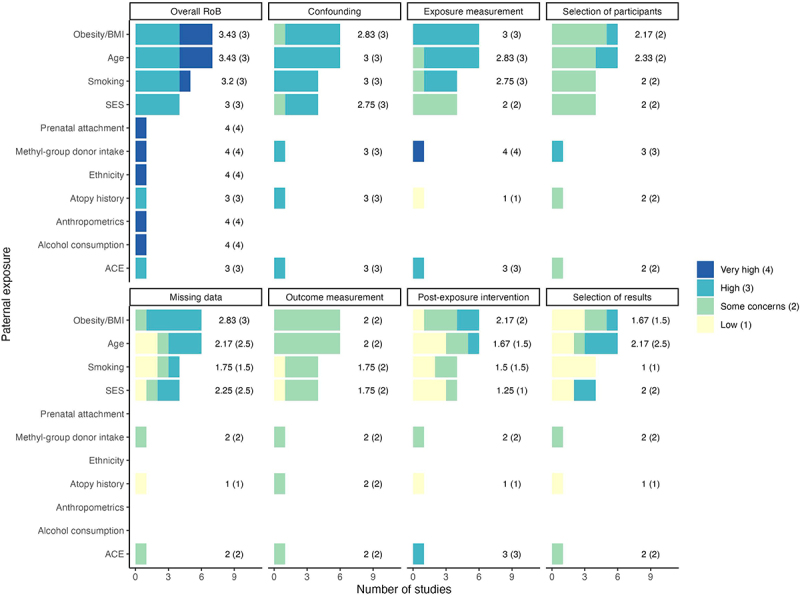
Distribution of risk of bias (RoB) stratified by exposure type. Mean and median of the RoB quantitative scores were displayed on top of each column. Very high = 4, High = 3, some concerns = 2, and Low = 1. BMI = body mass index; SES = socioeconomic status; ACE = adverse childhood experiences.

Studies that examined exposures such as smoking and SES scored relatively better overall across all domains (Figure 4). In particular, for exposure measurement, atopy history, SES, and age fared better than other exposures, with mean scores of less than three, as they were more likely to be constant within the exposure time window. Meanwhile, when looking at exposures related to health behaviors such as smoking, the extent to which the domain was biased depended mostly on whether the timing and dosage were well defined and measured accordingly. For example, looking at the four papers where smoking was the exposure of interest, three [41,43,44] were rated ‘high’ RoB, where one [58] was rated ‘some concerns’ (S4 Appendix: Excel Table S4). In this instance, the former papers showed mismatches between the hypothesized susceptible window, and timing and measurement of the exposure, contributing to the ‘high’ RoB rating, on top of this there were no specification of the hypothesized potential underlying mechanisms.

### Results of syntheses

All results from the review are summarized in Table 2 and organized by exposure type. Results from studies that were rated ‘very high’ RoB were excluded in the following discussion.

**Table 2.** Summary characteristics and results of the 28 studies included.

First author (year); final N	Paternal exposure	Exposure range (summary statistics) - only applicable for quantitative measure	Method of exposure measurement	Timing of paternal exposure measurement	Epigenetic marks (tissue)	Timing of offspring’s epigenetic mark measurement	Analytic approach (platform)	Main results	Overall RoB
Adkins *et al*. (2011); 89 mother-child pairs	Paternal age (continuous)	Range=18-39; mean=27; SD=5.1	Partner report	Postnatal	Methylation (cord blood)	Delivery	Genome-wide (27K)	No evidence of association	Very high
Atsem *et al*. (2016); 191 mother-father-child trios	Paternal age (continuous)	Range=26-55; median=37	Not reported	Not specified	Methylation (cord blood)	Delivery	Targeted [bisulfite pyrosequencing; deep bisulfite sequencing (verification)]	Inverse association between age and methylation at *FOXK1* and *KCNA7* DMRs; hypomethylation at *FOXK1* DMR was replicated in allele-specific verification for paternal allele	High
Potabattula *et al*. (2018); 39-46 mother-father-child trios	Paternal age and BMI (continuous)	Paternal age: Mean=38.5; SD=5.3 Paternal BMI:Mean=25.2; SD=2.7	Not reported	Not specified	Methylation (cord blood)	Delivery	Targeted (deep bisulfite sequencng)	Direct association between age and methylation at *MEST* (*PEG1*); allele-specific methylation (paternal allele).	Very high
Choufani *et al*. (2019); 88 placental samples, 44 samples from ART/infertile, 44 from spontaneously conceived	Paternal age (continuous)	Control group:Mean=36; SD=5.3	Not reported	Prenatal	Methylation (placenta)	Delivery	Genome-wide (450K)	No evidence of association	High
		ART group:Mean=37.4; SD=7							
Moore *et al*. (2019); 2740 women	Paternal age (continuous)	Not specified; only reported paternal age stratified by maternal age group	Child report	Offspring’s adulthood	Methylation (peripheral blood)	Adulthood (mean=57 years; range=35-75 years)	Genome-wide (450K)	17 differential methylated CpGs were identified below the genome-wide significance but CpG sites and estimates were not reported	High
								The residuals after regressing paternal age on maternal age were not associated with DNA methylation at those 17 CpGs	
Krug *et al*. (2020); 21 males of European origin (EWAS); 32 males (miRNA analysis)	Paternal age [young (22.7 years +/-1.9) vs. old (38.2 year +/-5.7)]	Mean=31.34; SD=5.54	Not reported	Offspring’s adulthood	Methylation; miRNA (peripheral blood)	Adulthood (individuals with young fathers=24.3±2.5 years; individuals with old fathers=23.8±3.1 years)	Genome-wide (450K); targeted	RNA expression (effect size was expressed in the form of mean transcript level relative to U6 small nuclear RNA):0.0012 +/- 0.0007 for young fathers; 0.0006 +/- 0.0003 for old fathers for miR-134	High
								EWAS: No evidence of association	
Andreu-Sanchez *et al*. (2022); 749 families	Paternal age (continuous)	Mean=46.01; SD=13.28	Not reported	Not specified	Methylation (peripheral blood)	Adulthood (43.9±13.7 years)	Genome-wide (450K)	Only presented genome-wide significant associations of methylations that resulted in the final 11 triplet associations for mediation; not all CpGs that reached genome-wide significance from EWAS were reported	Very high
								Reported CpGs included cg13107760 (ZNF804A), cg11041835 (*GULP1*), cg01959238 (*CTC*-786C10.1), cg05677025 (*CTD*-3080P12.3), cg13107760 (*ZNF804A*)	
								Inverse association between age and methylation at all CpG sites reported	
Hinz *et al*. (2012); 346 German/Caucasian mother-child pairs (healthy, term, and no low birth weight)	Paternal atopy history (ever vs. never; asthma, hay fever, atopic dermatitis)		Unclear	Prenatal	Methylation (cord blood)	Delivery	Targeted (methylation-specific PCR)	Lower Treg numbers in cord blood was associated with paternal asthma	High
Soubry *et al*. (2013); 67 families (IGF2 MDR) and 70 families (H19 DMR)	Paternal obesity [obese (BMI >=30 kg/m2) vs.non-obese]		Partner report	Not specified	Methylation (cord blood)	Delivery	Targeted (bisulfite pyrosequencing)	Binary and continuous BMI:	High
								Decreased methylation at *IGF2* DMR; increased methylation at *H19* DMR	
Soubry *et al*. (2015); 79 mother-child pairs	Paternal obesity [obese (BMI >=30 kg/m2) vs.non-obese; paternal BMI (continuous)		Partner report	Prenatal	Methylation (cord blood)	Delivery	Targeted (bisulfite pyrosequencing)	Binary: Decreased methylation at *MEST, NNAT, PEG3* DMRs	High
								Continuous: Decreased methylation at *MEST, PEG3* DMRs; increased methylation at *SGCE/PEG10* DMR	
Potabattula *et al*. (2018); 39-46 mother-father-child trios	Paternal age and BMI (continuous)	Paternal age: Mean=38.5; SD=5.3 Paternal BMI:Mean=25.2; SD=2.7	Not reported	Not specified	Methylation (cord blood)	Delivery	Targeted (deep bisulfite sequencng)	Direct association between obesity and methylation at *MEST* (*PEG1*); allele-specific methylation (paternal allele)	Very high
Noor *et al*. (2019); 429 father-mother-child trios (>= 34 gestation weeks, no pregnancy complications)	Paternal BMI (continuous)	Mean=26.4; SD=4.0	Partner report	Prenatal	Methylation (cord blood; buffy coat)	Delivery, early (median= 3.3; range=2.9-4.9) to mid-childhood (median=7.7; range=6.7-10.5)	Genome-wide (450K)	Birth:Decreased methylation at cg04763273 (*TFAP2C*) and increased methylation at cg17206978 (*CENPA*) after Bonferroni correction	High
								Decreased methylation at cg12837919 (*LSAMP*) and increased methylation at cg15687147 (*FAM190A*), cg19846622 (*MSX1*), cg26544752 (*CDH10*), cg07908498 (*SORCS3*), cg22355517 (*PDE3A*), cg01029450 (*ARFGAP3*) at FDR q<0.05 significance level	
								Age 3:Decreased methylation at cg04763273 (*TFAP2C*) and increased methylation at cg26544752 (*CDH10*), cg07908498 (*SORCS3*) at FDR q<0.05 significance level	
								Age 7: Decreased methylation at cg04763273 (*TFAP2C*) at FDR q<0.05 significance level	
								When stratified by maternal BMI > 25, 18 CpG sites were differentially methylated	
Potabattula *et al*. (2019); 113 father-child pairs	Paternal BMI (continuous)	Range=17.30–40.30; mean=25.78; SD=3.3	Measured	Preconception	Methylation (cord blood)	Delivery	Targeted (bisulfite sequencng)	No evidence of association	Very high
Prats-Puig *et al*. (2019); 63 father-child pairs	Paternal height, weight, BMI (continuous)	Paternal weight (kg):Median=80; IQR=75-86.2	Measured	Prenatal	Methylation; miRNA (placenta)	Delivery	Targeted (bisulfite pyrosequencing)	No evidence of association	Very high
		Paternal height (cm):Mean=176; SD= 6							
		Paternal BMI:Median=26; IQR=24.2-27.2							
Sharp *et al*. (2021); birth: 4894; childhood: 1982	Paternal BMI (z-scores; continuous)	Mean=26.98; SD=3.2	Self-report; partner report; calculated using height/weight measured	Time point as close to the time of pregnancy as possible	Methylation (cord blood; peripheral blood)	Delivery, early to mid-childhood (6.9±0.3 years)	Genome-wide (450K; EPIC)	No evidence of association for main analyses; 7 CpG sites were differentially methylated in female offspring at birth when stratified by sex	High
Joubert *et al*. (2014); 1035 mother-child pairs	Paternal smoking (yes vs. no)		Partner report	Prenatal	Methylation (cord blood)	Delivery	Genome-wide (450K)	No evidence of association	High
Bouwland-Both *et al*. (2015); 332 mother-father-trios	Paternal smoking (yes vs. no; among smokers, <5 cigarettes per day vs. >=5 cigarettes per day)		Partner report	Prenatal	Methylation (cord blood)	Delivery	Targeted (mass-spectrometry based bisulphite sequencing)	No evidence of association	High
Wu *et al*. (2019); 20 mother-child pairs (microarray methylation); 361 mother-child pairs (delivery); 211 mother-child pairs (6 years)	Paternal tobacco smoke (<20 cigarettes per day, > 20 cigarettes per day, without PTS exposure)		Unclear	Prenatal	Methylation (cord blood; peripheral blood)	Delivery; mid-childhood	Targeted [Illumina GoldenGate methylationPanel I array assay; bisulfite pyrosequencing (validation)]	Estimates were confusing to interpret and inconsistent with the definition of exposure mentioned in methods	Very high
Rauschert *et al*. (2019); 692 mother-child pairs	Paternal smoking (yes vs. no)		Partner report	Prenatal	Methylation (whole blood)	Adolescence (17 years)	Genome-wide (450K)	No evidence of association	High
Monasso *et al*. (2020); 938 father-child pairs	Paternal smoking (yes vs. no)		Partner report	Prenatal	Methylation (cord blood)	Delivery	Genome-wide (450K)	No evidence of association	High
King *et al*. (2015); Final MV model range from 458-573 for different DMRs	Paternal ethnicity (non-Hispanic black, Hispanic, Other and non-Hispanic white)		Partner report	Prenatal	Methylation (cord blood)	Delivery	Targeted (bisulfite pyrosequencing)	Models adjusted for maternal ethncity only:Decreased methylati on at *IGF2* and *MNAT* DMRs when compared black against white (ref) and hispanic against white (ref)	Very high
								Decreased methylation at *H19* DMRs when compared hispanic against white (ref) and other against white (ref)	
								Models further adjusted for SES-related covariates including maternal education and household income:Only the relationship with *IGF2* DMR in black vs. white (ref) persisted	
Lee *et al*. (2015); 164 mother-father-child trios	Paternal alcohol consumption (abstainer, light drinker, moderate drinker, heavy drinker)		Self-report	Prenatal	Methylation (cord blood)	Delivery	Targeted (methylation-specific PCR)	Decreased methylation at *SERT* DMR when compared father drinker/mother drinker against father drinker/mother non-drinker (ref) and increased methylation at *SERT* DMR when compared against moderate binge drinker against moderate non-binge drinker (ref)	Very high
								Decreased methylation at *DAT* DMR when compared against heavy binge drinkers against heavy non-binge drinkers	
Huang *et al*. (2016); 613 mother-child pairs (female offspring only)	Paternal SES [occupational class (6 categories: 1=highest/ professional to 6=lowest/manual; 2 categories: high=1–3 and low=4–6; education (years of education; 3 categories: <= 8 years, 9-12 years, >=13 years]		Partner report	Postnatal (1-2 days postpartum)	Methylation (peripheral blood)	Adulthood (mean age=32 years)	Targeted (mass-spectrometry based bisulphite sequencing)	Increased methylation at *ABCA1* gene region per unit increase of paternal occupational class; association became weaker when marginal structural models were used to account for time-dependent confounding	High
Alfano *et al*. (2019); 914 mother-child pairs (delivery); 973 mother-child pairs (age 7.5 years); 974 mother-child pairs (age 15.5 years)	Paternal SES (education: low, intermediate, high; occupation: manual vs. non-manual)		Self-report	Prenatal	Methylation (cord blood; buffy coat)	Delivery, mid-childhood (mean age=7.5 years), adolescence (mean age=15.5 years)	Genome-wide (450K)	No evidence of association with both paternal education and occupation in main analysis; sensitivity analysis additionally adjusting for maternal characteristics including age, pre-pregnancy BMI, smoking and alcohol consumption during pregnancy showed associations with paternal occupation in cord blood that reached FDR-corrected significance at cg20388823 (*GRID2*)- decreased methylation, cg11724366 (*FSTL3*)- increased methylation, and cg09655022 - increased methylation.	High
Joyce *et al*. (2021); CARDIA: Y15 (age 33-45 years; N=1089), Y20 (age 50 years; N=1092); FFCWS: Y9 (N=637), Y15 (N=609); Project Viva: Early childhood (N=120), mid-childhood (N=460); PROGRESS: Birth (N=948) and Y4 (n=948)	Paternal SES [education (US cohorts:< high school, >= high school, some college, >=college graduate; Mexican cohort: education in years); occupation (clerical/sales vs. executive/professional)		Self-report	CARDIA: Y0 FFCWS: postnatal Project viva: not mentioned PROGRESS: not specified	Epigenetic age acceleration (cord blood; peripheral blood; buccal swab)	Delivery, early (3-4 years) to mid-chilhood (7-9 years), adolescence (15 years), adulthood (33-50 years)	Genome-wide (EPIC; 450K); targeted (bisulfite pyrosequencing)	CARDIA Y15: increased epigenetic age acceleration (EAA) when compared less than high school against college of higher (ref) CARDIA generalized estimating equations (GEE) models combining both time points at Y15 and Y20 showed decreased EAA when compared some college against college graduate or higher (ref) and increased EAA when compared less than high school against college or higher (ref)	High
Simanek *et al*. (2022); 948 mother-child pairs	Paternal SES (education: <5 GCSE, >=5 GCSE, A-level, >A level or other); employment status: employed manual, employed non-manual, self-employed, uemployed or student)		Self-report	Prenatal	Methylation (cord blood)	Delivery	Genome-wide (EPIC)	No evidence of association with paternal education	High
								Increased methylation at cg22930484 when compared manual vs. non-manual paternal occupation	
Pauwels *et al*. (2017); 51 mother-father-child trios (healthy Caucasian mother, singleton)	Paternal methyl-group donor intake (continuous)		Self-report	Prenatal	Methylation; hydroxymethylation (cord blood)	Delivery	Global (UPLC-MS/MS); targeted (bisulfite pyrosequencing)	Increased methylation at *IGF2* DMR and global methylation per 100mg increase in betaine intake	Very high
								Increased methylation at CpG1 of *IGF2* DMR and mean CpG methylation at *IGF2* DMR per 100mg increase in methionine intake	
Pellicano *et al*. (2020); 13 mother-father-child trios (term, birthweight >2kg)	Paternal prenatal attachment and psychopathological symptomatology (continuous)		Self-report	Prenatal and postnatal (up to one month postnatal)	Methylation (buccal swab)	Early childhood (2 days; one month after delivery)	Global (dot blot assay)	Increased global methylation was associated with global severity index, somatization, depression, phobic anxiety, sleep disorders, and attachment quality at first month of life	Very high
								Decreased global methylation was associated with attachment quality, somatization, obsessive-compulsive, interpersonal sensitivity, depression, hostility, and sleep disorders between birth and first month of life	
								Increased global methylation was associated with global severity index and phobic anxiety between birth and first month of life	
								Increased odds in paranoid ideation and sleep disturbances when compared increased global methylation against decreased methylation	
Merrill *et al*. (2021); 45 father-child pairs	Paternal ACE (continuous)	Range=1-7; mean=1; SD=1.76	Self-report	Postnatal	Methylation (peripheral blood)	Early childhood (22.5±3.3 weeks)	Genome-wide (450K)	No evidence of association when looking at a priori candidate analysis (332 sites associated with child abuse identified based on 4 papers)	High
								Based on 96339 probes that were narrowed down using IQR filter to subset to only variable probes where the methylation beta value varies by at least 5% across samples in the 5th and 95th percentile:	
								Eight CpG sites were associated with paternal ACEs (continuous scores):	
								Decreased methylation at cg12030301 (intergenic), cg13615516 (intergenic), cg13688808 (*HCG4*), cg23505145 (*KLF1*), cg02380750 (intergenic)	
								Increased methylation at cg00049664 (*CMTM2*), cg10543947 (*APOL2*), and cg26297819 (*TEF*)	
								After dichotomizing paternal ACE to absence and presence of paternal ACEs, effect sizes at cg12030301 (intergenic), cg13688808 (*HCG4*), cg00049664 (*CMTM2*), cg26297819 (*TEF*) were attenuated (< biological threshold of |beta >0.03|)	

#### Age

Two studies [32,55] evaluated the association between paternal age and methylation changes at delivery using cord blood and placenta. Using methylation changes found in cord blood, Atsem *et al.* [55] found inverse associations between paternal age and methylation at forkhead box K1 (*FOXK1*) and potassium voltage-gated channel subfamily A member 7 (*KCNA7*) differentially methylated regions (DMRs) in bisulfite sequencing of targeted regions after multiple testing correction. Hypomethylation of *FOXK1* DMR was replicated in allele-specific verification for the paternal allele. *FOXK1* encodes a transcription factor that plays a role in the activation of myogenic progenitor cells and in skeletal muscle regeneration. According to the authors, *FOXK1* copy number variations (CNVs) have been previously associated with autism. By comparing *FOXK1* methylation levels in the peripheral blood of 74 autistic cases and 41 matched controls, they also found more profound decrease in *FOXK1* cord blood methylation in autistic individuals. Meanwhile, Choufani *et al.* [32] found no evidence of an association in a genome-wide analysis of placental methylation levels.

When measuring genome-wide methylation changes in peripheral blood in adulthood, Moore *et al.* [53] reported 17 differentially methylated CpGs (no reported CpG sites or estimates) after adjusting for estimated white blood cell proportions, the top six surrogate variables based on array control probes, experimental plate (94 samples per plate), participant age at blood draw, breast cancer status, and smoking status. However, the residuals after regressing paternal age on maternal age did not correlate with the 17 CpG sites. Krug *et al.* [57] reported no evidence of association, although methylation measurements were taken much later in the studies performed by Moore *et al.* [53] (mean of 57 years vs. mean of 24 years).

#### Atopy history

Offspring cord blood DNA methylation levels were used as a biomarker measure of immune function in a study performed by Hinz *et al.* [34] The Treg number was defined as the percentage corresponding to the measured amount of demethylation in the Treg-specific demethylated region (TSDR) in the forkhead box P3 (*FOXP3*) gene measured in cord blood at delivery. Comparing those with a paternal history of atopy (ever vs. never from asthma, allergic rhinitis, and atopic dermatitis) against those without, lower Treg numbers were found in the cord blood of children with fathers who had a history of asthma.

#### Obesity/BMI

Two studies from the same group [35,36] measured methylation in targeted gene regions in cord blood and found that obesity status and BMI were associated with methylation changes at multiple imprinted DMRs, specifically in those that were involved in early growth regulation. Comparing obese and non-obese fathers, decreased methylation at insulin-like growth factor 2 (*IGF2*), mesoderm-specific transcript (*MEST*), neuronatin (*NNAT*), and paternally expressed 3 (*PEG3*) DMRs, and increased methylation at the H19 imprinted maternally expressed transcript (*H19*) DMR were observed. Analyzing paternal BMI as a continuous variable, a higher BMI was associated with lower methylation of *IGF2*, *MEST*, and *PEG3* DMRs and higher methylation at *H19* and sarcoglycan epsilon/paternally expressed 10 (*SGCE/PEG10*) DMRs. Multiple testing corrections were not performed in these studies.

Two other studies [38,40] investigated the association between paternal BMI and genome-wide methylation across multiple time points from birth (cord blood) to childhood (peripheral blood). Noor *et al*. [38]. identified associations at two CpGs [transcription factor AP-2 gamma (*TFAP2C*) and centromere protein (A*CENPA*)] and seven CpGs [limbic system associated membrane protein (*LSAMP)*, the coiled-coil serine-rich protein 1 (*FAM190A*), msh homeobox 1 (*MSX1*), cadherin 10 (*CDH10*), sortilin-related VPS10 domain containing receptor 3 (*SORCS3*), phosphodiesterase 3A (*PDE3A*), and ADP ribosylation factor GTPase activating protein 3 (*ARFGAP3*)] at birth after Bonferroni correction and BH-FDR correction, respectively. Associations at three CpG sites (*TFAP2C*, *CDH10*, and *SORCS3*) were replicated at age three, and one site (*TFAP2C*) was further replicated at age seven, with a consistent direction of association. The CpG site, which was replicated across three time points, is located in the intron of AK097528, a cDNA clone generated from human testis. The closest gene, *TFAP2C*, is known to be an important developmental gene coding for transcription factor involved in cell pluripotency maintenance and craniofacial and skeletal development [61,62]. Eighteen CpG sites were differentially methylated when the analysis was stratified by maternal BMI greater than 25. Sharp *et al*. [40]. performed an EWAS meta-analysis using data from the PACE consortium, which included data from ten cohorts. No evidence of an association between paternal BMI and child DNA methylation (DNAm) was found in the main analysis at either birth or childhood (6.9 ± 0.3 years). Seven CpG sites were differentially methylated in female offspring at birth after stratification by sex. While being the largest paternal BMI-offspring methylation study to date, the authors did not replicate results from most of the candidate gene studies, as well as the CpG sites identified in the study performed by Noor *et al*. despite employing similar analytic strategies. Careful consideration of key study design elements, such as confounding structure and measurement error by Sharp *et al*. [40] contributed to more robust results among the studies.

#### Smoking

One study [41] that examined paternal preconception smoking reported by partners during pregnancy found no evidence of association with cord blood methylation using a genome-wide approach. Similarly, three studies [43,44,58] investigating partner-reported paternal smoking during pregnancy also did not identify evidence of paternal smoking being associated with methylation changes at delivery and age 17 years, in both genome-wide and targeted approach studies.

#### Socioeconomic status (SES)

All studies [47–50] on SES included education, occupation, or employment status as the SES indicators. Paternal education was not associated with methylation changes at delivery, mid-childhood, adolescence, or adulthood in the studies included in this review, except when methylation was included as a measure of epigenetic age acceleration (EAA) in adulthood [49]. However, there was evidence of an association with paternal occupation. Huang *et al*. [47]. found increased methylation in the ATP binding cassette subfamily A member 1 (*ABCA1*) gene region in adulthood (mean = 32 years) when ordinal occupational class categories were included linearly in their model. The *ABCA1* gene is functionally related to cholesterol homeostasis [63,64]. Simanek *et al*. [50]. also found increased methylation at one CpG site (cg22930484) in cord blood at delivery when they compared fathers with manual occupation with those who had non-manual jobs.

#### Adverse childhood experiences (ACEs)

In peripheral blood collected at age 3 months (22.5 ± 3.3 weeks), Merrill *et al*. [54]. found no evidence for association between ACEs and methylation at 332 CpG sites that had been previously associated with adverse childhood experiences in previous studies [65–68]. In a subset of 96,339 probes with a methylation beta value varying by at least 5% between the 5th and 95th percentiles, eight CpG sites were associated with paternal ACEs when analyzed as a continuous cumulative score.

## Discussion

### Main findings

In this systematic review, we included 28 studies that examined the association between preconception, prenatal paternal factors, and the offspring epigenome. Data sources mostly originated from the UK and the US, with little representation of the rest of the world. Obesity/BMI, smoking, and SES were commonly studied paternal factors and almost all (27/28) studies measured methylation as the epigenetic outcome of interest. Three studies additionally measured hydroxymethylation [51] and miRNA expression levels [39,57]. Most studies measured epigenetic outcomes at delivery (21/28), with the target tissue being almost exclusively cord blood. Very few studies have investigated timepoints beyond delivery, and if they do, blood remains the tissue of choice. Only half of the studies performed some form of adjustment for maternal effects, while the other half qualitatively compared paternal and maternal effect estimates. Approximately one-third of the studies have attempted functional analyses to understand the biological pathways involved in epigenetic outcomes. While validation was more commonly practiced among the studies in our review, most of them discussed their findings in the context of previous literature instead of performing actual analyses, and only one study [40] tried to replicate their findings in other cohorts and performed a meta-analysis of those results. When assessing the overall strength of evidence of paternal factors on epigenetic patterns in offspring, studies were at ‘high’ or ‘very high’ RoB, mainly due to issues in confounding, exposure measurement, missing data, and selection of participants. We found that results varied across studies and there was little to no overlap in CpG sites or genomic regions in most of these methylation studies by exposure type except for smoking and paternal education, which showed a consistent lack of evidence of association with the offspring epigenome in our review.

### Comparison to the literature

To our knowledge, this review is the first to evaluate the strength of evidence linking preconception and prenatal paternal factors to the offspring epigenome. A previous systematic review [24] examined the associations between environmental factors and DNA methylation in human spermatozoa in men but not in their offspring, concluding there was a lack of overlap in findings with the same or related questions, even among high- and moderate-quality studies.

Among the three studies [69–71] included in the review [24] investigating sperm DNA methylation differences between smokers and non-smokers, while two [69,71] identified CpG regions related to protein tyrosine kinase genes, none reported changes in the same genomic regions. In contrast, we found no evidence associating paternal smoking during preconception or prenatal periods with offspring DNA methylation measured in cord blood at delivery or in peripheral blood during adolescence. Most studies relied on maternal reports about paternal smoking during pregnancy, except one [41] that considered preconception smoking measured during pregnancy but did not clearly define the preconception window, asking the mothers whether their partners had smoked before pregnancy. A recent study [72] (published after our search date) using data from the Respiratory Health in Northern Europe, Spain, and Australia study (RHINESSA) found associations between paternal smoking (preconception and pubertal onset smoking <15 years) and offspring blood methylation in individuals aged 7 and 50. The authors defined preconception smoking as smoking that started before childbirth minus 2 years, and measures were self-reported by fathers. They found more pronounced effects in CpGs that were associated with pubertal onset smoking. However, these findings were not replicated in the Avon Longitudinal Study of Parents and Children (ALSPAC) cohort, which measured blood methylation at ages 15 to 17 years. The authors suggested that the lack of replication may have been due to low statistical power, as consistent directional effects were observed. Another study [73] using the same RHINESSA data and the European Community Respiratory Health Survey (ECRHS) data found associations between paternal smoking and offspring DNA methylation in 195 individuals (ages 11–54 years). This study was excluded from our review because the window of exposure was indicated as the offspring’s childhood, which was outside the window of exposure we were interested in. However, since the sample included fathers who started smoking preconceptionally in RHINESSA, the associations might have been driven by these individuals. The genes annotated to the CpGs and DMRs differed in the two studies, but some were involved in similar pathways related to innate immunity. This suggests that that paternal preconception smoking, particularly during early pubertal onset, may influence offspring epigenetic pathways related to innate immunity, while paternal prenatal smoking does not. Rodent studies [74] that exposed male mice preconceptionally to cigarette smoke, have similarly shown an over-representation of the immune response – related transcripts in the prefrontal cortex in the offspring of male mice exposed to cigarette smoke. However, cigarette smoke-associated changes in paternal sperm methylation did not overlap with those in the offspring’s prefrontal cortex or with sperm of unexposed offspring. The authors suggested that DNA methylation may serve as a marker rather than a mechanistic driver of intergenerational effects, with other epigenetic mechanisms such as chromatin status and small ncRNAs (sncRNAs) potentially playing a more direct role.

Previous studies [47,48,75] have consistently shown associations between prenatal maternal education and offspring methylation changes. However, we did not identify evidence relating prenatal paternal education to offspring blood methylation at delivery, mid-childhood, adolescence, or adulthood, except when epigenetic age acceleration in adulthood was included as the outcome. The discrepancies observed between maternal and paternal education levels were consistent with those observed with paternal and maternal BMI [40]. This indicates that paternal factors may influence offspring epigenetics via mechanisms different from maternal factors, other than methylation, or in different tissue types. We could not identify studies that examined paternal education and epigenetic outcomes in parents themselves. Our previous study [14] showed paternal education was consistently and more strongly associated with maternal pregnancy biomarkers than maternal education, suggesting paternal factors may influence offspring health through maternal pathways. Additionally, sperm epigenetics have been hypothesized to affect offspring health through inheritance theory [76]. Hence, maternal and paternal epigenetic outcomes are important alternatives to consider alongside offspring epigenetic outcomes. The lack of evidence may also reflect limited statistical power, as detecting paternal effects requires a larger sample size than maternal effects.

Paternal obesity or BMI was the most studied paternal exposures in relation to the offspring epigenome in our review. Yet, reviewed studies suffered from confounding and exposure measurement error, contributing to inconsistent findings. Differences in paternal obesity and BMI observed in non-experimental studies may have arisen from various mechanisms including over- or under-nutrition, exercise, weight training, pharmacology, or metabolic pathophysiology and may also be acute or long-term conditions. Consequently, individuals with similar high (or low) BMI may have different underlying physiology with corresponding differential impacts on paternal physiology, sperm epigenomics, and fetal programming. Therefore, it is not surprising that the offspring epigenetic signals found in this review were not generally reproducible due to the ill-defined exposures. Rodent models that use well-defined diet-induced obesity manipulations (*i.e*., high-fat diet (HFD)) with clearly specified dose, timing, and duration of exposure consistently demonstrate that paternal HFD results in epigenetic changes in offspring sperm [77–79] and somatic tissues such as liver [77], adipose tissue [77], and pancreatic islet [11], including alterations in DNA methylation and sncRNA expression. Sperm DMRs in these F1 offspring were shown to enrich in genes that are involved in the cellular processes, while changes in sncRNAs were shown to alter the gene expression enriched in metabolic pathways [11,77]. It was also observed that altered gene expression in offspring tissues can occur in the absence of corresponding differential DNA methylation [11,77], suggesting gametic DNA methylation differences are either erased during reprogramming or do not persist long-term in somatic tissues, and that transcriptional changes may be mediated by alternative developmental and epigenetic mechanisms.

### Interpretation of findings

Literature examining the biological links between paternal factors and offspring outcomes has only recently emerged. Historically, paternal influences on child health has been overlooked in biological science, but recent efforts have been invested in understanding the role of phenotype inheritance through sperm or seminal plasma, especially when considering peri-conceptional factors [4,24]. Apart from the direct genetic transmission of alleles, other proposed mechanism involves the transmission of epigenetic factors via sperm, such as DNA methylation, ncRNAs, and chromatin modifications [76]. However, a systematic review [24] of DNA methylation in human spermatozoa found limited and conflicting evidence regarding its impact on pregnancy outcomes and offspring health, even in imprinted genomic regions believed to escape epigenetic reprogramming.

Animal studies have explored various epigenetic mechanisms, including DNA methylation [11,77], sncRNAs [11,77], histone methylation [80], and mitochondrial tRNAs [81] across both parent and offspring germ cells and somatic tissues, to explain how paternal environmental exposures may be transmitted. Techniques like zygotic microinjection of sperm contents [11,82] have strengthened the evidence surrounding sncRNAs as the potential drivers of epigenetic inheritance, particularly when transcriptional changes are reflected in offspring target tissues without corresponding DNA methylation changes [11,77]. However, human studies have lagged in exploring these alternative epigenetic mechanisms, limiting our understanding of the intergenerational transmission of paternal effects in humans.

While animal studies demonstrate that sperm-mediated epigenetic inheritance is a plausible mechanism for transmitting paternal effects, the social influences that fathers have on mothers during preconception and pregnancy, as well as paternal parenting and intergenerational transmission of behaviors [83–85], can also affect offspring phenotype and potentially shape the epigenome. This multiplicity of pathways suggests that merely finding associations between paternal factors and the offspring epigenome is insufficient to distinguish between these putative mechanisms in human studies. Compounding this challenge, many human studies rely on suboptimal observational data, with paternal factors often poorly defined, misclassified or assessed at an etiologically ambiguous or less relevant times such as postnatally. Consequently, studies assessing paternal effects on offspring epigenetics and health more broadly are highly disparate, with a generally high likelihood of bias. In contrast, animal studies typically involve controlled settings, where interventions are well-defined and introduced before conception, allowing researchers to exclude potential postnatal and social effects, ultimately isolating pathways involving paternal germline-mediated epigenetic inheritance.

To improve the quality of human studies on paternal effects on the offspring epigenome, emulating conditions similar to those in an ideal experiment could be beneficial. One promising approach is ‘target trial emulation,’ a framework developed to help researchers design and analyze human observational studies as if they were randomized controlled trials [23]. This framework emphasizes the importance of clearly defining specific exposures, limiting measurements and follow-ups to etiologically relevant time points, and carefully addressing missing data and confounding factors, all significant challenges to human studies of paternal effects. These principles are captured in the risk of bias tool, ROBINS-E [22]. It has been argued that a better implementation of these standards can address the most pressing biases that threaten the evidence syntheses of causal effects in molecular epidemiology [86]. We demonstrated the use of ROBINS-E [22], developed based on the target trial framework, by focusing on paternal factors as the exposure of interest and epigenetic changes as the outcome, a largely understudied area for perinatal and child health. Although there are limitations associated with the tool [87], we found it valuable in assessing key design issues that are commonly faced in human observational studies. We provide some evidence that human studies analyzed in this way are also more likely to recapitulate animal model findings, strengthening confidence in their conclusions.

A notable challenge in human studies of paternal effects is the absence of a clearly defined research hypothesis that involves biologically plausible mechanisms within etiologically relevant exposure window. Previous studies, such as those performed by Moore *et al.* [53], Rauschert *et al.* [43], and Monasso *et al.* [44], have treated paternal factors as secondary to maternal factors without exploring the mechanisms specific to paternal effects. For instance, in Monasso *et al*.’s study on maternal smoking and offspring DNA methylation [44], the analysis of paternal smoking was restricted to CpG sites associated with maternal sustained smoking. While the authors cited limited statistical power as a reason for their analytical strategy, they did not explore any plausible biological mechanisms for justification nor perform verification and validation for paternal associations [44]. Many studies in our review also hypothesized that epigenetic mechanisms mediate paternal influences on offspring health. Yet, few studies performed formal mediation analysis or appropriately accounted for maternal effects. Evidence from animal studies demonstrates that including both paternal and offspring epigenetic changes – not just DNA methylation but also other alternative epigenetic mechanisms such as sncRNAs or histone modifications – as well as other omics data can help clarify the mechanistic links between paternal exposures and epigenetic changes. Moreover, maternal effects should be appropriately accounted for either by including maternal factors as covariates for adjustment, effect modification, or mediation to isolate paternal contributions. Hence, formulating a well-defined research hypothesis that includes specific biologically plausible mechanisms distinct from maternal effects is not only crucial, but also beneficial for planning statistical strategies that are concordant with the hypothesis.

Since epigenetic outcomes exhibit more variability and are less stable than genotypes, precise exposure measurements aligned with relevant windows are essential for improving the interpretability of epigenetic studies. Human observational studies often suffer from poorly defined exposure measurements including timing, type, and dose, as described in the case of paternal BMI/obesity. In order to reliably interpret differential methylation in obese versus non-obese fathers as causal effects of obesity per se, formal causal inference literature requires us to justify that the effect of moving anyone from non-overweight to overweight status (or vice versa) is the same or ‘consistent’ on average for everyone in the target population [88,89]. This is clearly violated if we consider a general population might include a mix of individuals who are normal or underweight due to chronic disease or after surgical or pharmacological weight loss, or technically overweight due to high muscle mass, or simply those who have been overweight for decades versus those who might be more transiently overweight. Violations of this consistency assumption also complicate the identification of confounders needed to achieve exchangeability (*i.e*., no unmeasured confounding), leading to potential residual confounding. Moreover, many birth cohort studies often lack preconception exposure data for fathers, leading most studies to rely on retrospective or partner reports collected during pregnancy. This often results in unwanted recall or selection bias, particularly when there is a discrepancy between the susceptible windows of interest and the timing of measurements. The extent of bias also depends on whether the exposure is likely to remain constant or varies over time, and the specificity regarding the length of the exposure window.

As the critical window of susceptibility for males can begin as early as in utero during primordial germ cell (PGC) reprogramming and extend through gamete maturation during each reproductive cycle in adulthood [90], the preconception period for males may span their entire life course. However, the preconception period is rarely properly defined for males, resulting in poor data measurement, as observed in our review. For example, following our pre-registered protocol, we excluded 12 studies due to incorrect paternal exposure measurements that were taken during the postnatal period and two additional studies where the timing of measurement was ambiguous. These studies lacked clarity regarding the relevant exposure windows they aimed to investigate and might have been intending to examine preconception effects. They represent a quarter of potentially eligible published studies on the subject and are another example of the challenges of synthesizing evidence from studies that do not properly define their exposure window of interest.

Nonetheless, exposures that are more likely to be constant, such as paternal education, are less susceptible to bias and tend to yield more consistent results, as indicated in our review. In contrast, exposures that are likely to vary over time can become problematic, when the duration of the exposure window extends over a long period. Research has shown that health behaviors, such as smoking and alcohol consumption, change as women enter pregnancy [91,92]. While relatively little research has been conducted on changes in health behavior in fathers as they transition to fatherhood, a recent study indicated that this transition was associated with weight gain and lower self-reported health [93]. Longitudinal approaches that account for time-varying exposures and confounding, such as marginal structural models [94], should be considered if exposures vary over time. These models can provide insight into the persistence, attenuation or potential reversibility of epigenetic effects throughout the life course.

Here, we illustrate a simple example of emulating a ‘target trial’ to understand the effect of paternal BMI change through physical activity on offspring epigenome at birth using observational data. The target trial would focus on a healthy subpopulation of men (without metabolic or fertility complications, not engaged in a physical activity program in the past six months, planning conception within a year). Such a target trial would compare those who voluntarily participated in a structured three-month preconception physical activity program (consisting of 60 minutes of resistance and aerobic training three times a week) with those who did not exercise, and follow them from the start of intervention to the birth of their child. With a well-defined question, exposure, timing, and length of follow-up, we can then define a set of alternative explanations to rule out or investigate as potential mediators or effect modifiers. These may include thinking about all the potential mechanisms through which an ‘effect’ could be transmitted, as comprehensively covered above, and the type of data to be analyzed including paternal and maternal germline epigenotype, and gene expression data in relevant tissues. Appropriate statistical methods can then be chosen based on those clearly defined parameters and data structure. Compared to studies relying solely on paternal BMI, this target trial-based study – characterized by precise definitions of population, exposure, dose, timing, and outcome – can produce more robust and interpretable evidence, even when using observational data without randomization. Aligning epigenetic measurements with developmentally relevant windows and tissues further enhances the potential for meaningful mechanistic insight and interpretability.

### Strengths and limitations

This review subjected the current area of research that is understudied – paternal and child health – to the highest rigor of observational causal inference using ROBINS-E [22]. Our review offers insights into the existing gaps in the current state of research and the methodology used in epigenetic epidemiology. These include the fact that poorly defined hypothetical mechanisms and exposures (*i.e*., lack of consideration in timing, dose, and time-varying components) make consistency and exchangeability difficult to justify, leading to quantitative estimates with no clear interpretation or potential validity. However, there are some limitations to our study, including the likelihood of non-paternity, which could introduce bias to the results. Some exposures had only one study included in the review, and thus no comparison could be made for a more insightful discussion. Additionally, we might have missed studies that defined their exposure window vaguely using words like ‘early childhood’ or ‘childhood’ to encapsulate exposures that were measured before delivery when screening the abstract. We only focused on investigating the key study design parameters that might generate bias in the current studies of paternal exposure and offspring epigenome by implementing the ROBINS-E tool, as it has previously been shown that a good study design helps facilitate appropriate downstream analyses and increases the chance of providing biologically interpretable results. However, there are challenges in applying the ROBINS-E tool. While the risk of bias ratings may be strict for assessing confounding, they were also more generous in certain aspects where non-reporting was under-penalized compared to papers that did report relevant information. As previously pointed out by Bero *et al.*, [87] we also found it challenging to differentiate studies with different biases, where studies may be rated as having the same overall risk of bias. However, to gauge how biased the studies were across domains, we assigned scores to the different ratings to obtain summary estimates. Despite these limitations, ROBINS-E offers the convenience of a checklist to help reviewers consider critical issues in interrogating evidence robustness and reproducibility using a methodologically sound approach.

## Conclusion

In our systematic review evaluating the association between paternal factors and the offspring epigenome, we found little to no overlap in CpG sites or annotated genomic regions for each exposure type. Most studies were rated ‘very high’ and ‘high’ risk of bias. Studies were particularly threatened by the poor definition of the research questions and exposure. Given the myriad potential mechanisms linking paternal exposure to the offspring epigenome, better attention to exposure definition, including dosage, timing of exposure, and issues of consistency as indicated by the target trial framework should be followed. This will support better reporting and interpretability of epigenetic studies and help distinguish plausible mechanisms and the types and timing of paternal interventions that might be supported by the data to improve offspring health.

## Data Availability

Availability of data and materials Supplementary file S4 can be accessed at doi:10.6084/m9.figshare.29205044.
